# Invasive brown treesnakes (*Boiga irregularis*) move short distances and have small activity areas in a high prey environment

**DOI:** 10.1038/s41598-022-16660-y

**Published:** 2022-07-26

**Authors:** Scott M. Boback, Melia G. Nafus, Amy A. Yackel Adams, Robert N. Reed

**Affiliations:** 1 Department of Biology, Dickinson College, Carlisle, PA USA; 2 Fort Collins Science Center, U.S. Geological Survey, Fort Collins, CO USA; 3 Pacific Island Ecosystems Research Center, U.S. Geological Survey, Hawaii National Park, HI USA

**Keywords:** Ecology, Behavioural ecology, Conservation biology, Invasive species

## Abstract

Animal movements reflect temporal and spatial availability of resources as well as when, where, and how individuals access such resources. To test these relationships for a predatory reptile, we quantified the effects of prey abundance on the spatial ecology of invasive brown treesnakes (*Boiga irregularis*) on Guam. Five months after toxicant-mediated suppression of a brown treesnake population, we simultaneously used visual encounter surveys to generate relative rodent abundance and radiotelemetry of snakes to document movements of surviving snakes. After snake suppression, encounter rates for small mammals increased 22-fold and brown treesnakes had smaller mean daily movement distances (24 ± 13 m/day, $\overline{x }$ ± SD) and activity areas (5.47 ± 5 ha) than all previous observations. Additionally, snakes frequenting forest edges, where our small mammal encounters were the highest, had smaller mean daily movement distances and three-dimensional activity volumes compared to those within the forest interior. Collectively, these results suggest that reduced movements by snakes were in part a response to increased prey availability. The impact of prey availability on snake movement may be a management consideration when attempting to control cryptic invasive species using tools that rely on movement of the target species to be effective.

## Introduction

Animals move to access critical resources distributed across the landscape. These movements are the result of a dynamic interplay of internal (e.g., internal state, locomotion, and navigation capacities) and external factors (e.g., biotic and abiotic threats, food availability, and spatial distribution of resources^1^). Moving can require a significant energetic investment and can increase an individual’s exposure to potential threats. Hence, animals are predicted to respond to variation in resource density and distribution by adjusting movement frequency and/or distances to balance benefits with costs^2^. Therefore, knowledge of animal movements provides important insights into the ecology of individuals, populations, and ecosystems^3^. Further, because conservation and management tools frequently rely on predictable animal movements^4,5^, understanding the factors that drive space use will also assist efforts to conserve species.

One of the salient features influencing animal movement patterns is food availability. Much of our understanding of how animals adjust movement in response to changes in food availability comes from food supplementation studies^6–8^. Most of these have observed reduced movement frequency, home range size, or other spatial parameters in response to high food availability^6,7^. However, many of these investigations focused on birds and mammals. Food supplementation studies using reptiles are comparatively few and of those, results are mixed. Within snakes, a handful of studies have examined movement patterns in response to food supplementation—all of these studies focused on vipers^9–11^ and involved offering prey directly to snakes in the field. However, food supplementation studies are not feasible for all snake species and, in bringing food to snakes, obviate the need for movement devoted to predatory behavior^12^. A single study investigated movements of a colubrid snake (the brown treesnake, *Boiga irregularis*) in response to experimentally decreased prey in the field^13^. These authors demonstrated increased movement rates, dispersal distances, and activity areas in response to reduced rodent availability^13^. Additional experimental manipulations of prey populations in situ are necessary to understand the influence of prey availability on snake movement.

From a management perspective, movement frequency is a critical parameter because it is often positively correlated with detectability^14,15^. Many animals reduce activity to avoid detection by predators^2,16^ and those colonizing a novel environment can reduce movements due to fear of novelty or neophobia^17^. Such reductions in activity can create a challenge for land managers attempting to develop management plans where movement patterns are critical, especially for cryptic species^4^. Therefore, movement ecology studies incorporating motivations for movement are vital for such conservation decisions. In this study, we investigated the movement ecology of the invasive brown treesnake (*Boiga irregularis*; hereafter BTS) in an experimentally manipulated population at low density and with high prey availability.

The brown treesnake is a highly cryptic, semi-arboreal snake that uses a mixture of active and ambush foraging tactics^18^. BTS invaded the island of Guam shortly after World War II^19,20^ where it caused the local extinction of at least 15 vertebrate species^19,20^. Due to their high abundance and ability to stow away in shipping containers, there is a high risk that BTS could become established on nearby snake-free islands^21^. Currently, BTS are managed on Guam using snake traps, toxic bait, and visual surveys^22^. The success of these control tools is partially dependent on detectability, a parameter influenced by a number of environmental parameters including snake activity^23–25^. Although BTS movement ecology was investigated previously on Guam^26–29^, all of those studies were performed in locations with presumably suppressed prey populations; much lower than prey abundance on neighboring snake-free islands^30–32^. Therefore, understanding BTS movement patterns in areas with high prey availability may facilitate our understanding of how snakes may respond during an accidental introduction to novel areas with prey availabilities higher than those on Guam.

In this study, we used radiotelemetry to examine daily movement distances, activity areas, and perch heights of BTS in a suppressed population with high prey availability. By combining radiotelemetry of BTS and visual surveys of small mammals, we aimed to determine (1) the response of a major prey category (small mammals) to snake suppression, (2) BTS movement ecology in a location with high prey availability, and (3) the role of additional factors such as snake sex and size on BTS movements. Results from this study may help managers predict movement behavior of BTS that are accidentally transported to other Pacific islands including the nearby Commonwealth of the Northern Mariana Islands and thereby help promote informed control and containment efforts.

## Results

### Small mammal catch per unit effort (CPUE)

Suppression of BTS appeared to result in irruption of small mammal populations in the Habitat Management Unit (HMU). Before snake suppression, we encountered 13 small mammals in 575-person hours of transects for a CPUE of 0.023—small mammals encountered/survey-person hours. After snake suppression, we encountered 222 small mammals in 456-person hours of transects for a CPUE of 0.487. Thus, we detected 22 times more small mammals after BTS suppression relative to before suppression (Table 1). After snake suppression and during the BTS telemetry, small mammal CPUE on the edge transects was higher (2.3 ± 0.7 small mammals/hr), compared to interior transects (0.8 ± 0.2 small mammals/h, *df* = 8, *T* = 5.8, *p* < 0.001).

**Table 1:** Visual survey results of small mammal encounters in the Habitat Management Unit (HMU) before snake suppression (March 2010–September 2012 and March 2013–August 2013) and after snake suppression (31 May 2015 to 6 August 2015).

	Survey location	Searcher nights	Searcher distance (km)	Searcher hours	*Suncus murinus*	*Mus musculus*	*Rattus* sp.	CPUE all small mammals
Before snake suppression	Edge	132	116.6	284.8	0	2	10	0.042
	Interior	119	116.0	290.5	1	0	0	0.003
							Total	0.023
After snake suppression	Edge	80	67.0	152.4	53	0	50	0.676
	Interior	154	133.7	303.8	17	0	102	0.392
							Total	0.487

Catch per unit effort (CPUE) is defined as total small mammals encountered/survey-person hrs.

### BTS movement in a prey rich environment

**Figure 1:**
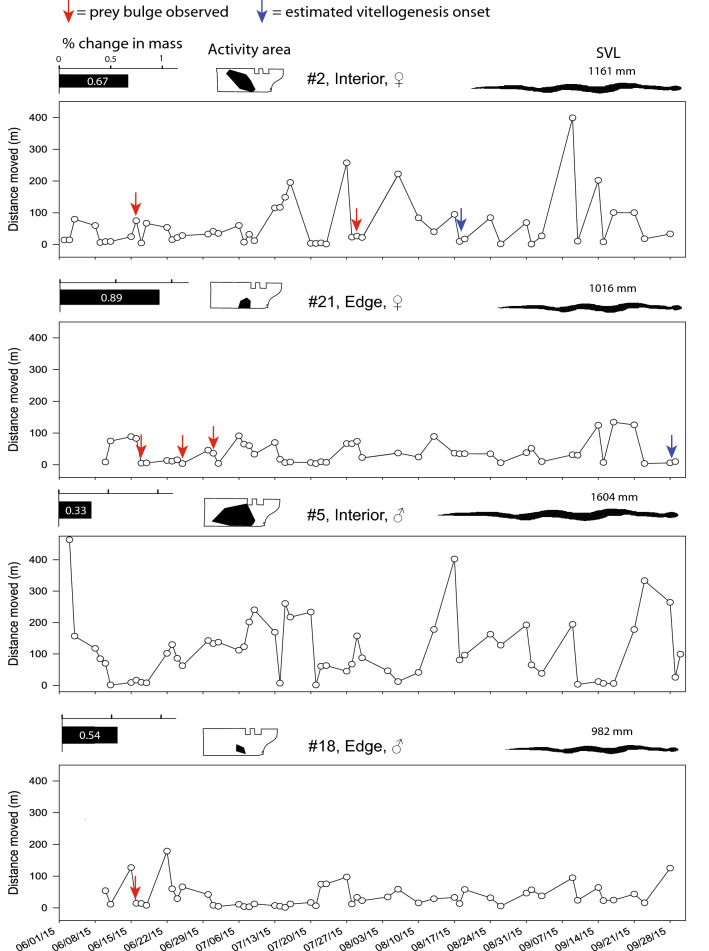
Movement patterns (average linear distance moved per day) on Guam of four radiotracked BTS in this study. Within each plot, we have indicated times when a prey bulge (indicative of prey consumption) was observed (red arrows) and, for females, estimated onset of vitellogenesis (blue arrows, estimated using follicular growth data obtained from Mathies et al.^33^). At the top of each movement plot is each snake’s % change in body mass (relative to mass at the time of transmitter implantation, precise value indicated as white text within bar), their activity area (minimum convex polygon [MCP] method) within the Habitat Management Unit (HMU, perimeter of HMU indicated as black outline), and their size in snout-vent length (SVL, relative size indicated by black snake silhouette with precise numerical value in mm above).

**Figure 2:**
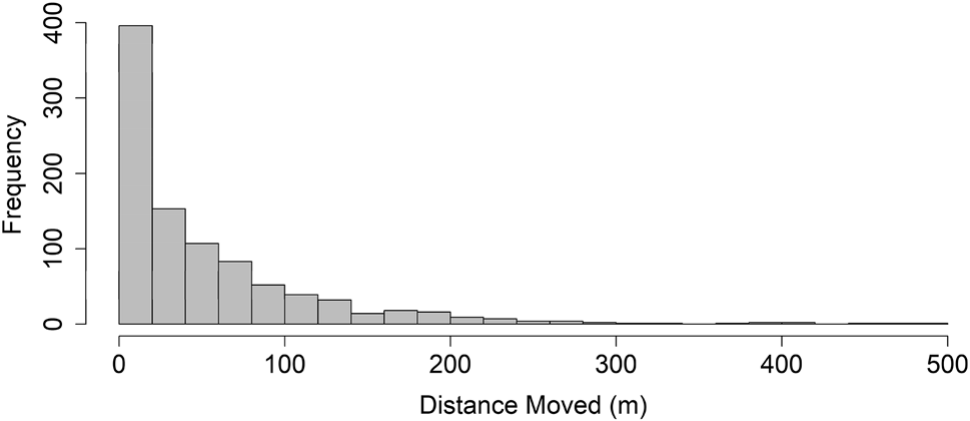
Frequency distribution of average distance moved (straight-line distance between successive locations/number of intervening days) per day for radiotracked brown treesnakes (BTS) in this study. The distribution was significantly right-skewed (Skewness = 2.55, SE of Skewness = 0.08).

**Figure 3:**
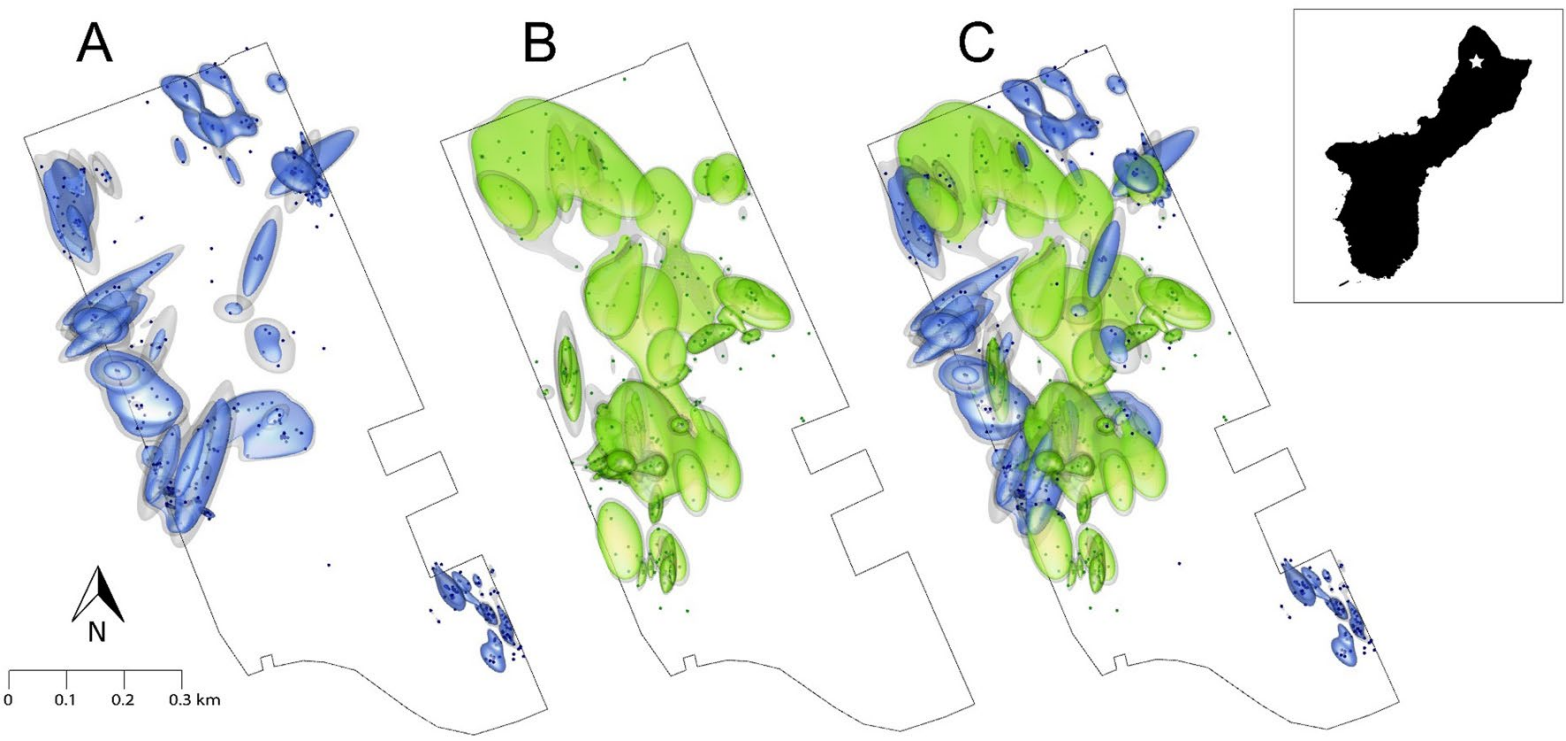
Three-dimensional activity volumes (3D Kernel Utilization Distributions, KUDs) of radiotracked brown treesnakes (BTS) frequenting the forest edge (**A** blue 75% isopleths surrounded by grey 95% isopleths), the forest interior (**B** green 75% isopleths surrounded by grey 95% isopleths), and combined (**C**) on Guam. Edge snakes (those with > 30% [mean = 63 ± 22%] of observations within 50 m of forest edge) had significantly smaller 3D activity volumes compared to interior snakes (those with < 30% [mean = 13 ± 7%] of observations within 50 m of forest edge; *df* = 18, *T* = − 2.29, *p* = 0.04). Outer black line in each panel represents the Habitat Management Unit (HMU, 13.596 N, 144.865 E) boundary fence. Inset shows the relative position of the HMU (indicated by the star) on the north end of Guam.

Telemetered snakes moved on average 24 ± 13 m per day (median = 20.4, 95% confidence limits = 17.7–29.5, Table S1) and had a mean activity area of 5.47 ± 5 ha (minimum convex polygon [MCP] method, Table 2). Snakes generally moved in a fluctuating pattern consisting of sporadic long-distance moves (> 100 m) followed by several days of short distance moves (Fig. 1). A frequency distribution of movement distance was significantly right-skewed (Fig. 2, Skewness = 2.55, SE = 0.08). We observed noticeable prey bulges in almost half (9/20) of telemetered snakes after snake suppression (13 total observations, Table S2). Most (75%) snakes exhibited reduced movement (< 40 m per day) for two or more days after we first observed a prey bulge (Fig. 1; Table S2). Additionally, snakes that primarily used forest edge, where small mammal CPUE was greatest, tended to move shorter distances (17.1 vs 26.4 m) per day (*df* = 18, *T* = − 2.13, *p* = 0.05) and had smaller 3D activity volumes (2.6 vs 9.7 ha^3^) compared to snakes with activity areas in the forest interior (*df* = 18, *T* = − 2.29, *p* = 0.04; Fig. 3).

**Table 2:** Average (± SD) two-dimensional (minimum convex polygon (MCP) and 2D Kernel Utilization Distribution (KUD) and three-dimensional (3D KUD) activity areas for brown treesnakes (*Boiga irregularis*) in the Habitat Management Unit (HMU) during high prey availability.

	This study			Santana–Bendix 1994	Christy et al. 2017	
	Male *n* = 9	Female *n* = 11	Sexes combined *n* = 20	Sexes combined *n* = 11	Treatment *n* = 12	Control *n* = 12
MCP (ha)	5.1 ± 7	5.8 ± 4	5.47 ± 5	20.8 ± 9(SE)	89.9 ± 18	99.2 ± 10
2D KUD (ha)	4.1 ± 5.5	3.3 ± 2.4	3.7 ± 4.0			
3D KUD (ha^3^)	7.2 ± 9.6	7.8 ± 6.3	7.54 ± 7.7			

Activity areas from other studies are included for comparison. For the Christy et al. 2017 reference, Treatment = prey suppression plots created by applying rodenticide prior to monitoring, Control = plots not treated with rodenticide.

**Figure 4:**
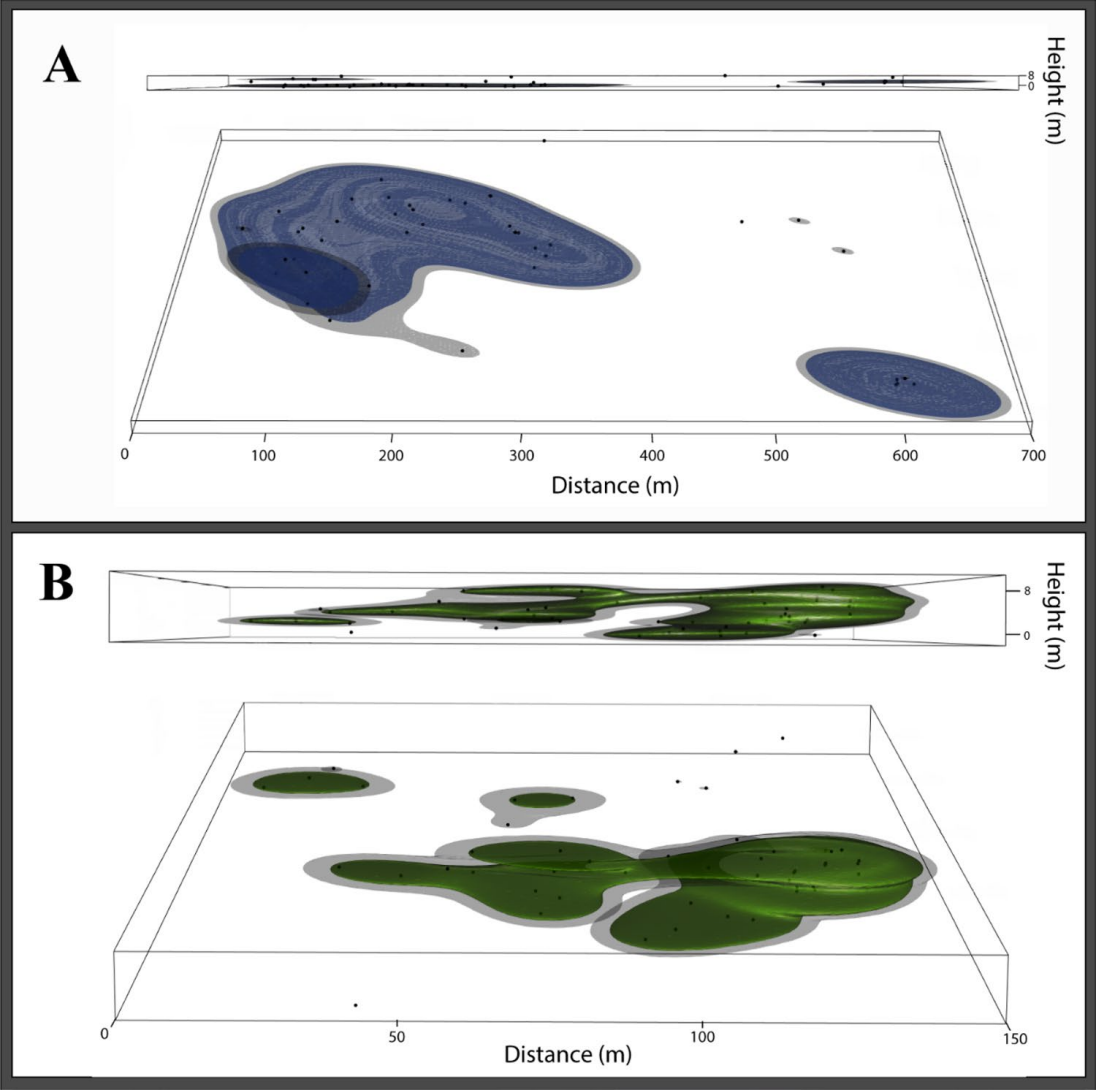
Examples of large (18.53 ha^3^) and small (3.06 ha^3^) 3D activity volumes from two male brown treesnakes (*Boiga irregularis*) with 1820 mm snout-vent length (SVL) (**A**, blue 75% isopleths surrounded by grey 95% isopleths) and a 982 mm SVL (**B**, green 75% isopleths surrounded by grey 95% isopleths). Two plots are shown in each panel, one from the lateral perspective as if you were standing on the ground (height = 0) and the other from approximately 45° angle from above. Note how both 3D activity volumes approximate a series of oblate spheroid shapes.

Three-dimensional activity volumes of BTS approximated oblate spheroids or a series of oblate spheroid shapes (Fig. 4 and Videos S1 and S2) with aspect ratios (*E*) less than 0.2 (average *E* = 0.04 ± 0.04). These extremely flat activity volumes indicate the vertical distances traversed by snakes were miniscule compared to the horizontal distances. Light Detection and Ranging (LiDAR) data revealed the canopy height averaged 12.2 ± 2.9 m (range 0–23.3 m) in the HMU but this varied across the site. The highest canopy occurred near the southcentral portion and the shortest canopy at the northern portion of the HMU (Figures S1 and S2).

### Movement based on sex and size

On average, males moved 27.7 ± 17 m per day while females moved 20.3 ± 7 m per day; this difference was not significant (*df* = 18, *T* = − 0.32, *p* = 0.75). Regardless of sex, larger BTS moved greater distances between tracking events relative to smaller snakes (*df* = 18, *T* = 5.54, *p* < 0.001), such that for each mm increase in snake size, distance traveled increased by 0.05 m/day. There was no difference between sexes in the total size of either 2D activity areas (*df* = 18, *T* = − 1.86, *p* = 0.08) or 3D activity volumes (*df* = 18, *T* = − 1.84, *p* = 0.08; Table 2). Regardless of sex, larger snakes had larger 2D activity areas (*df* = 18, *T* = 4.71, *p* < 0.001) and 3D (*df* = 18, *T* = 2.74, *p* = 0.01) activity volumes.

Females were underground (13% of 572 relocations) significantly more often (Fisher’s Exact Test: *df* = 1; *p* < 0.001) than males (4% of 394 relocations). Interestingly, females were only slightly less visible to researchers (44 ± 12%) compared to males (47 ± 11%) and this difference was not significant (Fisher’s Exact Test: *df* = 1, *p* = 0.07). Irrespective of sex, snakes in better body condition at the start of the study were more likely to use underground retreats (*R* = 0.48, *p* = 0.03).

### Perch height

**Figure 5:**
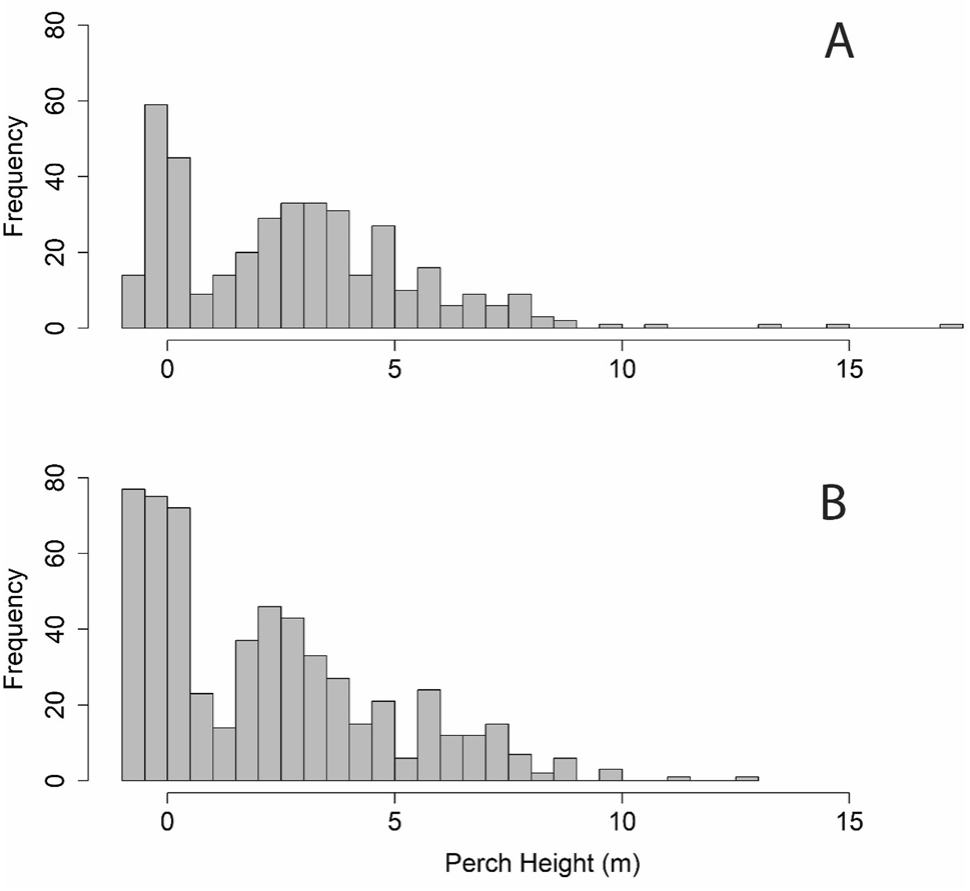
Frequency distribution of perch heights (m) for radiotelemetered male (**A**, *n* = 9) and female (**B**, *n* = 11) brown treesnakes (*Boiga irregularis*) after snake suppression within the Habitat Management Unit (HMU) on Guam. Notice the difference in the frequency of heights < 0 (underground) between the sexes reflecting an increased use of underground retreats by females.

Male and female snakes did not differ in their perch heights (*df* = 18, *T* = 1.39, *p* = 0.18) although there was much individual variation in this parameter. Males were found on average 2.93 ± 2.7 m from the forest floor whereas females averaged 2.33 ± 2.7 m. Further, there was no effect of snake size (SVL) on average perch height (*df* = 18, *T* = − 1.25, *p* = 0.23). Frequency distributions of perch heights for male and female snakes were both bimodal and right-skewed (Fig. 5). However, a Kolmogorov–Smirnov Test revealed a significant difference between the two distributions (*D* = 0.123, *p* = 0.002) which may have been due to increased use of subterranean retreats by females (Fig. 5).

### Body condition and reproduction of BTS

Of the sixteen telemetered snakes recovered at the end of the study (9F, 7 M), 94% (15 of 16) had gained mass and nearly all females (8 of 9) had developed vitellogenic follicles (> 5 mm). Based on published rates of follicular development in BTS^33^, the onset of vitellogenesis for 7 of 8 females was during September 2015 while one female that contained shelled eggs likely began vitellogenesis in late August.

## Discussion

Our study is the first to document movement patterns of BTS within a reduced-density predator population with high prey (small mammal) availability on Guam. As a first step in this investigation, we documented changes in relative prey abundance in our study site after suppression of the BTS population. Post-toxicant visual surveys for BTS revealed a decrease (up to 79%) in the number of snakes within the HMU^34^. In the current study, visual surveys for small mammals conducted five months after snake suppression revealed a 22-fold increase in small mammal encounters relative to before snake suppression. BTS in this relatively high-prey environment moved shorter distances and had smaller activity areas relative to all other populations studied to date^13,26,27^. We hypothesize that our telemetered snakes responded to high prey availability by feeding frequently and decreasing their postprandial activity, which resulted in reduced daily movements and small activity areas. Below we explore the evidence that supports this hypothesis.

We believe our CPUE estimates accurately reflected some level of increased small mammal availability for the following reasons. First, small mammals are known to have small home ranges and achieve extremely high densities, especially on islands^30,35^. Second, population estimates based on encounter surveys can accurately reflect population size in both birds and mammals^36,37^, and incidental observations also provide accurate estimates of small mammal abundance^38^. Lastly, a small mammal trapping study, conducted independently of ours but temporally and spatially concurrent, demonstrated an increase in rat (*Rattus diardii*) capture rates after snake suppression relative to those obtained prior to snake suppression^39^. Thus, based on this evidence, we believe our estimates portrayed a considerable increase in small mammal abundance after snake suppression that could have affected movements of surviving BTS during our study.

The sharp increase in the abundance of small mammals directly followed BTS suppression and was presumably due to a top-down effect of predator reduction^40,41^. With the exception of the well-documented extirpations of native vertebrates on Guam by brown treesnakes^19^, studies on the effect of ectothermic predators on their respective prey populations are limited but there is some evidence that these species can and do limit vertebrate prey populations^42–44^. Augmenting adder (*Vipera berus*) populations on two islands in the Baltic Sea resulted in decreased Field Vole (*Microtis agrestis*) density, but relative to controls this difference was not statistically significant^45^. In another field experiment, researchers introduced large (max SVL 107 mm) curly-tailed lizards (*Leiocephalus carinatus*) to small islands in the Bahamas where they prey on the smaller (max SVL 57 mm) brown anole (*Anolis sagrei*)^46^. Within two months of introducing curly-tailed lizards, brown anole populations decreased by approximately 50% and this effect persisted for two years until populations of both species were exterminated by a hurricane^46^. The abundance of a skink (*Carlia ailanpalai*) and two gecko species (*Lepidodactylus lugubris* and *Hemidactylus frenatus*) roughly doubled on plots on Guam after experimentally removing BTS^44^. Thus, it appears that ectothermic predators in general, and snakes in particular, can control prey populations^47^.

After snake suppression, surviving BTS were at a lower density and were presumably exposed to high prey availability. Daily movement distances of BTS in this low predator density, high prey environment were shorter than those reported in other studies of BTS on Guam^26–28^. These previous studies differ from ours in several ways and therefore comparisons to our results should be interpreted with caution. For instance, some of these studies estimated animal locations by triangulation from pre-established stations thus introducing greater error^26,27^ and, in most cases, were conducted in different habitats or targeted smaller snakes^28^. Moreover, our snakes were contained within a barrier whereas other studies have not had similar spatial constraints for reasons described below. Nevertheless, some comparisons can be made. Tobin et al.^27^ tracked snakes in a secondary forest near our study site and during a similar seasonal period as our study yet reported daily movement distances (64.4 m/day) greater than ours (23.6 m/day, 95% CI [17.7, 29.5), a difference which may reflect, in part, the higher tracking frequency used in the Tobin et al. study (multiple times in 24 h) relative to our study (2 to 4 days/week). Further, the frequency distribution of daily movement distances for BTS in our study was strongly right skewed with a median of 20 m/day indicating most movements were much shorter than the mean would suggest.

Although snakes in our study were contained within a barrier fence, we do not believe the barrier was a significant factor in limiting movements among our telemetered BTS. For instance, while many of our snakes frequented the edges of the HMU, we witnessed only two occasions (out of 970 relocations) in which snakes scaled the fence. Further, although we did not monitor snakes continuously, apart from one female BTS, snakes did not exhibit movements that suggested they contacted the fence more than once. Several appeared never to contact the fence, and all failed to move along the fence as they might have if trying to move around it. Thus, BTS in our study moved short distances, a behavior that does not seem to be a result of the barrier fence.

Evidence from other studies demonstrates a negative correlation between prey availability and snake movements. For instance, racers (*Coluber constrictor*) at a South Carolina, USA wetland exhibited greater daily movements (3 times longer) and larger home ranges (6 times greater) relative to populations of the same species in Kansas and Utah^48^. These authors attributed differences in snake movements to a long-term drought in South Carolina that occurred during their study and that likely resulted in a decrease in prey availability. Similarly, Madsen and Shine^49^ not only demonstrated that water pythons (*Liasis fuscus*) increased movements when prey became scarce but also exhibited long-distance movements that closely tracked the seasonal migrations of their major prey, dusky rats (*Rattus colletti*). While these studies establish correlations between changes in prey availability and snake movements, in our study we increased small mammal abundance by experimentally suppressing the predator (snake) population, which allowed us to examine how BTS might alter their movements in response to these changes.

The strongest support for a direct effect of prey abundance on BTS movement ecology comes from a study with a manipulation opposite of ours. Using a rodenticide, Christy et al.^13^ experimentally suppressed the rodent population in replicate plots while leaving control plots untreated. In rodent-suppressed plots, BTS had greater dispersal distances, greater movement rates, and larger activity areas relative to snakes on control plots^13^. Estimated activity areas of BTS from their control and rodent-suppressed plots were much larger than we found in the HMU (and larger than other studies estimating BTS activity areas^26^). Differences in activity areas may have had more to do with differences in habitat between the studies rather than prey densities. Christy and colleagues^13^ performed their field manipulation in southern Guam, a habitat dominated by grasses and shrubs while our study occurred in a secondary limestone forest with an average canopy height of 12.2 m. Nevertheless, decreased movement distances and activity areas in plots with higher rodent density (control plots) is consistent with our hypothesis that small activity areas and relatively short movements of BTS in the HMU were direct responses to high prey abundance.

BTS activity areas in the high prey environment were smaller than those reported in all previous investigations^13,26^. This is more remarkable considering that our activity area estimates were potentially biased larger than their true size because we did not bound our estimates at the perimeter fence. If this bias had strongly influenced our conclusions, we might expect activity area estimates for edge snakes to be larger than estimates for interior snakes. However, we observed precisely the opposite; edge snakes had activity areas that were smaller than those of interior snakes (see more on this in Edge Effect below). Thus, while activity areas for some of our snakes were likely biased large, we do not believe this played a big part in our interpretations.

Interestingly, 3D activity volumes were oblately spheroid in shape. Aspect ratios for BTS activity volumes were extremely low, less than 0.2, indicating they were flat in shape. To visualize this, an aspect ratio of 0.2 for a 3D activity volume would be analogous to the Shanghai Ferris Wheel in Shanghai, China laying on its side with a diameter of 100 m and a maximum center height of only 4 m. However, such a flat 3D activity volume might be expected for an arboreal snake considering the limits of forest structure. For instance, arboreal animals are comparatively restricted in their vertical movements, even in forests with high canopies, relative to their horizontal movements^50^. Indeed, canopy heights within the HMU closely matched the perch heights selected by BTS. Therefore, flattened 3D activity volumes of BTS were more likely the result of vertical habitat limits and less likely the result of snake behavior. Interestingly, sea snakes (*Hydrophis* sp.) navigating aquatic environments relatively free from such vertical limits exhibit more globose 3D activity volumes^51^.

Previous work supports the idea that frequent prey consumption can affect movement patterns of snakes, but results are mixed. We know of three studies where snakes were supplementally fed in the wild and their movements compared to control (unfed) snakes^9–11^. Only two of these studies found differences between groups. First, Wasko and Sasa^10^ found that supplementally-fed terciopelos (*Bothrops asper*) exhibited shorter and less frequent movements but had similar sized home ranges relative to unfed snakes. Second, Glaudas and Alexander^11^ found that supplementally-fed puff adders (*Bitis arietans*) moved shorter distances and spent less time foraging, but did so within similar sized home ranges compared to unfed snakes. Hence, supplemental feeding of vipers appears to decrease average daily movements but not necessarily activity areas/home ranges.

Behavioral observations of our telemetered snakes also suggest they ate frequently and exhibited reduced movement for some time after eating. For instance, we encountered nine of our 20 snakes with noticeable prey bulges on 13 occasions. Most (75%) of these snakes moved shorter distances (< 40 m) for two or more consecutive days after observing a prey bulge. These patterns are consistent with other studies that found detectability of BTS fluctuated on a 4–7-day cycle^23,25^. Further, our telemetered snakes contained large fat reserves and had high body condition when necropsied at the end of the study. Snakes in better body condition were more likely to use underground retreats relative to those in poorer condition and may have selected such retreats to safely digest prey. However, females were relocated underground 13% of the time compared to 4% for males (this discrepancy was also revealed by differences in perch height distributions of male and female BTS) and most females (8 of 9) became vitellogenic during the study. Thus, subterranean retreats may have been driven by a demand for water retention during reproduction^52^. Nevertheless, behavioral and morphological observations are consistent with the hypothesis that frequent feeding on small mammals could be responsible for decreased movements of our telemetered BTS.

Forest edges affect organisms due to a variety of biotic and abiotic conditions inherent to these regions^53^. Temperature^54^, light exposure^54,55^, tree density^56^, and plant species richness^57^ can be greater at the forest edge whereas air moisture^54^, soil moisture^55^, and canopy cover^58^ tend to be lower at the edge. Identifying relevant factors influencing BTS and how they utilize forest edges is not straightforward; snakes could benefit from one or a combination of factors and could be affected directly or indirectly. For instance, BTS may be attracted to forest edges due to a direct effect of increased solar exposure for increased basking opportunities. Alternatively, increased plant species richness and/or productivity could indirectly affect BTS by improving conditions for their prey. Studies have demonstrated forest edges can support a high abundance of small mammals^59^ and some species of birds select edge habitats for food or shelter^60,61^. Black ratsnakes (*Pantherophis* [*Elaphe*] *obsoletus*) actively selected edge habitats during the bird nesting season^62,63^. On Guam, the spatial distribution of endothermic prey relative to forest edges is unknown.

Our telemetered snakes with activity areas along the forest edge had smaller 3D activity volumes and moved shorter distances relative to those in the forest interior. We also recorded the greatest increase in small mammal encounters along forest edges, although rat encounters were also high in the forest interior. Snakes use multiple sensory modalities to locate prey^64,65^ and may have selected edge habitats as good locations to locate prey. Laboratory and field experiments on BTS demonstrated that the combination of olfactory and visual cues created the strongest orientation response compared to either of these cues alone^66^. Among our telemetered snakes, we did not see evidence of directional movements towards the forest edge, but this may have been due to the frequency at which we tracked snakes (2–4 days/week) or because snakes had already established activity areas in/near edge habitats.

Lastly, it is important to consider the limitations of our study. First, our estimates of abundance for predator (BTS) and prey (small rodents) were based upon visual encounter surveys rather than the more rigorous methods of mark-recapture and associated statistics^37^. Second, while we believe the data reported herein are sound, we concede that some of our comparisons could have been limited by the number of animals we could effectively track. Further, because this snake population had previously been subjected to toxic bait application, we must consider the possibility that survivors exhibiting reduced movement distances were those that refused toxic prey and that toxicant application may have selected for snakes with individual traits^67^ that contributed to the observed effects. Hence, although we report a compelling correlation between increased prey abundance and predator behaviors, additional data are needed before we can establish causation.

## Conclusion

Our study is the first to describe movement patterns of a snake predator in a low-density population exposed to high prey availability. Suppressing this population appears to have led to a transient increase in prey abundance that influenced the movement behavior of surviving snakes. The bottom-up effect of rodent abundance on BTS spatial ecology mirrors a manipulative study performed on a smaller scale with replicate controls^13^ and is consistent with studies demonstrating a negative relationship between movement distances and feeding rates in snakes^10,11,25^. Additionally, increased small mammal encounters observed immediately after the snake suppression augments evidence that ectothermic predators regulate prey populations^44,46^. Importantly, our findings suggest this invasive snake could exhibit markedly reduced movements in habitats outside of Guam where prey densities are much higher^30^ and where there is a real threat of establishment^21,68,69^. This suggests that eradication of incipient populations may require a major labor investment that warrants continued development of detection tools for snakes at low densities and/or occupying high prey environments.

## Methods

### Study site

We conducted the study within the Habitat Management Unit (HMU) located on Andersen Air Force Base (AAFB) on the northern end of the island of Guam (13.596 N, 144.865 E; Figure S3). The HMU was completely enclosed with a snake-exclusion barrier that prevented snake immigration into the HMU but allowed emigration. The barrier was comprised of 3.6 km of a 1.8-m high chain-link fence covered with 6-mm galvanized steel mesh that formed an overhang or bulge on the exterior that prevented snakes from climbing over^70^. The interior area of the HMU was 55 hectares (length of longest side = 1300 m, width = 480 m).

The forest within the HMU consists of a disturbed limestone forest with an overstory dominated by *Vitex parviflora*, a non-native species. The nearly monotypic stand of *Vitex* is broken up with patches of Coconut (*Cocos nucifera*) and a scattering of native canopy trees throughout (e.g., Fagot, *Ochrosia oppositifolia*, Banyan, *Ficus prolixa*, Yoga, *Elaeocarpus joga*, and Breadfruit, *Artocarpus mariannensis*). Some portions of the HMU retain a mixed native mid- and understory including Screwpine (*Pandanus tectorius*), Paipai (*Guamia mariannae*), Mapunyao (*Aglaia mariannensis*), Sea hibiscus (*Hibiscus tiliaceus*), and Pawpaw (*Carica papaya*). The understory in some parts of the HMU, especially the southern third, is dominated with a monoculture of the native fern, *Nephrolepis hirsitula*^70^ (Figure S4).

Over the course of 16 months, the HMU was subjected to aerial applications of baits toxic to BTS (dead juvenile mice affixed with 80 mg of acetaminophen^22,71^). Eight applications occurred between 3 September 2013 and 19 December 2014 for a total of 15,840 baits in 16 months^71^. Successful suppression of BTS was supported by decreased bait take rates (bait tube monitoring^39,71^) and reduced BTS encounters during visual surveys^34^. BTS encounters dropped below baseline (pre-toxicant) levels shortly after the first toxicant application and remained low for up to 10 months after the last toxicant application was completed^39^. Based on catch per unit effort (CPUE, defined as the number of BTS encountered/survey-person hours), surveys for BTS revealed a 65% decrease in snake encounters of all sizes classes (CPUE before suppression = 0.699, CPUE after suppression = 0.248) and a 79% decrease in large BTS (> 900 mm SVL) relative to baseline levels (CPUE of large BTS before suppression = 0.247, CPUE of large BTS after suppression = 0.053)^34^. Hereafter, we refer to the pre-toxicant period (18 May–28 October 2010 and 26 March–13 August 2013) as “before snake suppression” and the post-toxicant period (31 May through 28 September 2015) as “after snake suppression”.

### Radiotelemetry methods

Five months after the last toxicant application, we implanted radio transmitters into 20 BTS (> 76 g) in the HMU. Thirteen of the transmittered snakes were collected within the HMU and seven were collected outside of the HMU, (hereafter referred to as translocated; translocation distances ranged from 5 to 15 km [mean = 9.2 ± 4 km]), in part to obtain large individuals (> 900 mm SVL) suitable for transmitter implant.

Radiotransmitters (model PD-2, 3.8 g; Holohil Systems Ltd., Canada) were surgically implanted following the procedures of Reinert and Cundall^72^ and did not exceed 5% of the snake’s mass. After capture, snakes were maintained in a climate-controlled facility at the Guam National Wildlife Refuge (GNWR) with water provided ad libitum. Snakes held for more than five days were offered a small, dead mouse equivalent to 10% of the snake’s body mass every 5 days. No snake was fed in the seven days prior to or after transmitter implantation. Male snakes (*n* = 9) averaged 1274 ± 306 mm snout-vent length (SVL) and 388.5 ± 367 g and were slightly larger than females (*n* = 11) that averaged 1051 ± 97 mm SVL and 168.5 ± 87 g (*df* = 7, *T* = − 1.98, *P* = 0.04). After surgery, snakes recovered in captivity for 2–3 days, after which time HMU-resident snakes were released at their point of capture and translocated snakes were released near the center of the HMU.

Using headlamps, radio receivers (Communications Specialists, R-1000), and directional antennas (Telonics, 2-element H-style), a crew of four biologists worked in pairs to locate all snakes between 1900 and 2400 h; each pair of biologists tracked approximately half the snakes (*n* = 10) each night. To avoid disturbing snakes, locations of hidden/inactive snakes were determined to within a two-meter area. Snakes were tracked four times per week between 31 May–06 Aug 2015 (resulting in 648 relocations) and two times per week for an additional seven weeks between 10 Aug–28 Sep 2015 (for an additional 322 relocations). Based on variation in transmitter battery life and some snake mortality, periods of tracking ranged from 52 to 142 days per snake (mean = 107 ± 24 days) and we relocated snakes between 25 and 61 times (mean = 48 ± 9, Table S1).

At each snake relocation, we recorded the snake’s position using a Garmin 60CSx GPS (± 5 m accuracy), noted the absence or presence of a visible prey bulge (indicative of recent feeding), and visually estimated the snake’s perch height in the forest to the nearest 0.5 m. Snakes detected on the ground received a perch height score of zero and those that were below ground and not visible were assigned an arbitrary value of − 1 m.

To evaluate body and reproductive condition of snakes at the end of the study, recovered snakes (*n* = 16) were euthanized, weighed, and necropsied. We calculated body condition as the ratio of snake mass to the snake’s expected mass given its length (expected mass estimated from mass × SVL regression based on more than 10,000 snakes sampled throughout Guam^23,34^). We documented and measured all follicles greater than 5 mm, the size at which follicles mature and become vitellogenic in BTS^73,74^. By comparing follicle sizes of our BTS with data on follicular development in BTS^33^, we estimated the onset of vitellogenesis for all of our female snakes that contained enlarged follicles.

### Visual surveys for small mammals

As part of a larger study to understand the efficacy of visual surveys of BTS at low densities^34,75^, we conducted nocturnal visual surveys for small mammals before (March 2010–September 2012 and March 2013–August 2013, average of 2.96 km of transects/night) and after snake suppression (31 May 2015–6 August 2015, average of 5.28 km/night). Using headlamps and working in teams of two, four biologists (independent from radiotelemetry teams) surveyed established transects, searching for small mammals and BTS (ten interior transects [forest on both sides] and eight edge transects [forest on one side]; Supplemental Figure S3). Surveys began no earlier than 30 min after civil twilight, with approximately equal time devoted to interior and edge transects. We recorded all small mammals (*Rattus* sp., *Suncus murinus*, and *Mus musculus*) encountered during surveys. For analysis, we combined data into a “small mammal” classification because BTS are known to consume all three species we observed^76^ and we had no reason to believe BTS prefer one species over another. We calculated the small mammal CPUE before and after snake suppression and separately for interior and edge transects. This allowed us to estimate relative availability of prey at two times and two locations.

### Analysis

Our spatial analyses focused on mean daily movement distances (straight-line distance between two successive locations divided by the number of intervening days, hereafter movement distance), perch height, and activity area. To characterize movement distances, we report mean, median, and a frequency distribution for these data. Distributions were considered significantly skewed if the absolute value of skewness divided by the standard error of skewness was greater than 2.0^77^. Because BTS are semi-arboreal and can spend considerable time in elevated positions in the forest^18^, we estimated activity spaces in both two-dimensions (minimum convex polygons [MCP] and kernel utilization distributions [2D KUD]) and in three-dimensions (3D KUD^78^). Using the package *ks* in program R^79^, we calculated 2D and 3D KUDs for each of our telemetered snakes using code adapted from^80^ and^51^. We used a plug-in bandwidth selector to estimate the smoothing factor that has been found reliable in estimating animal ranges^81^. We then rendered the 3D KUDs by utilizing the *rgl* and *misc3d* packages in R. We calculated volumes at both 75th and 95th isopleths^82^ and used these in visualizing activity volumes of each snake. We used 95th isopleths (contours) for all statistical analyses, hereafter referred to as 3D activity volumes.

To characterize the overall shape of a snake’s 3D activity volume we first assumed the ranges approximated spheroid shapes and then calculated their aspect ratios (*E*). Aspect ratio is defined as the ratio of a spheroid’s width (major axis) to its height (minor axis): *E* = 1 for perfect spheres, *E* < 1 for oblate spheroids (i.e., pancake shaped), and *E* > 1 for prolate spheroids (i.e., vertical rod shaped). We selected maximum horizontal distance between any two vertices within their convex polygons to represent the major axis of the 3D activity volume while the difference between the maximum and minimum perch height represented the minor axis.

To characterize the vertical space available to snakes within the HMU, we generated a Canopy Height Model using LiDAR data obtained from NOAA (https://coast.noaa.gov/htdata/lidar1_z/geoid12b/data/4939/) in ArcGIS Pro. To do so, the first and ground returns were isolated and converted into two raster digital elevation models (DEMs) with one-meter grids. Ground returns were subtracted from the first returns to generate canopy heights and the model was clipped to the study area. Using the spatial analyst toolbox, we calculated summary statistics along three transects through the study site (two along the edges and one through the middle of the HMU, Figure S1).

To examine the effect of forest edge on snake movement ecology, we created a value of the relative “edginess” for each snake. For the purposes of our linear models (see below) we defined edginess as a continuous variable of the relative amount of a snake’s activity area (2D KUD) that occurred within a 50-m buffer around the HMU fence. We defined forest edge as the space between the HMU fence and 50 m into the forest because previous studies of BTS found 50 m to be the maximum penetration of an edge effect^53^. To visualize potential differences in snake activity areas between animals that frequented forest edge versus forest interior, we divided snakes into equal groups: edge was individuals with > 30% of their locations within the 50-m buffer while interior was individuals with < 30% of their locations within the 50-m buffer.

We performed three linear models examining the effects of SVL, sex, and edginess on four response variables: 2D activity area (2D KUD), 3D activity volume (3D KUD), mean distance moved per day, and mean perch height. Initially, we modeled whether snakes were translocated into the HMU or were a resident because this variable failed to improve model fit, and because preliminary comparisons between these groups failed to indicate differences in space use^34^, we omitted resident status from ensuing analyses. We compared all possible candidate models with different combinations of our independent variables and implemented the model with the lowest Akaike Information Criterion (AIC) values. All response variables (prior to analysis) were square-root-transformed to meet assumptions of linearity except 3D activity volume which was log-transformed. Post-hoc pairwise comparisons of coefficients were conducted, and *T*-values and associated *P*-values are reported.

We used a Fisher’s Exact Test to compare proportions (e.g., visibility of snakes, use of underground retreats), and we used a Pearson’s correlation when comparing the relationship between body condition and frequency of underground refuge use. The R programming environment (version 3.4.0^79^) was used for all statistical tests with additional spatial analyses performed in ArcGIS 10.3.1^83^ including calculation of MCPs. Dispersion about means is indicated with ± 1 SD unless otherwise indicated and α was set at 0.05 for all tests. Research involving animal subjects was approved by the USGS-FORT Institutional Animal Care and Use Committee (FORT IACUC 2015-08). All experiments were performed in accordance with these guidelines and the authors complied with ARRIVE guidelines.

## Supplementary Information

Supplementary Information.

Supplementary Video S1.

Supplementary Video S2.

## Data Availability

Datasets generated and analyzed during this study are available in two USGS data releases via the online digital repository ScienceBase.gov: 10.5066/P939BM0W and 10.5066/P95QJ2PE^75,84^.
