# Supplementary material for: Invasive brown treesnakes (Boiga irregularis) move short distances and have small activity areas in a high prey environment
Source: Sci Rep. 2022 Jul 26;12:12705. doi: 10.1038/s41598-022-16660-y (PMC9325984; doi:10.1038/s41598-022-16660-y)
Supplement: Supplementary file 1 — Supplementary Information. [file 41598_2022_16660_MOESM1_ESM.docx]

**Supplemental File Legends:**

Video S1. Animated Graphics Interchange Format (GIF) of rotating 3D activity area (3D KUD, blue 75% isopleths surrounded by grey 95% isopleths; total volume = 3.15 ha^3^) for a female brown treesnake (Snake 21) with an SVL of 1016 mm. We observed evidence of prey consumption (presence of a prey bulge) three times for this individual and, by the end of the study, the snake had increased 1.8 times its original mass.

Video S2. Animated Graphics Interchange Format (GIF) of rotating 3D activity area (3D KUD, pink 75% isopleths surrounded by grey 95% isopleths; total volume = 8.13 ha^3^) for a female brown treesnake (Snake 1) with an SVL of 1150 mm. We observed evidence of prey consumption (presence of a prey bulge) once for this individual and, by the end of the study, the snake had increased 0.5 times its original mass.

Figure S1. Canopy height (m) profiles obtained from LiDAR data for three transects from eastern edge (A), middle (B), and western edge (C) of the Habitat Management Unit (HMU) on Guam. Left panel shows the outline of the HMU (dark black line) with the three transects indicated with thin black lines. Note that the for the profile graphs, the x-axis differs slightly due to the irregular shape of the southern end of the HMU.

Figure S2. Canopy height surface model (A) generated from LiDAR data for the Habitat Management Unit (HMU) using ArcGIS Pro. Using the trend tool in the Spatial Analyst Toolbox, we generated first (B), second (C), and third (D) order polynomial trend surfaces to illustrate trends in canopy height across the site. LiDAR data obtained from: <https://coast.noaa.gov/htdata/lidar1_z/geoid12b/data/4939/>

Figure S3. Map of the Habitat Management Unit (HMU) located on Andersen Air Force Base on the northern end of the island of Guam (13.596 N, 144.865 E) generated using ArcGIS 10.3.1 (ESRI 2017, <https://www.esri.com/en-us/arcgis/about-arcgis/overview>). Interior transects (*n* = 10) were 440 meters in length and are indicated with blue lines while edge transects (*n* = 8) were 220 meters and are indicated with orange lines.

Figure S4. Limestone forest structure on southern end of the Habitat Management Unit (HMU) site. Note the dense understory of the native fern, *Nephrolepis hirsitula* that occurs in tree canopy gaps in this portion of the forest. Emergent/canopy trees in the background are *Vitex parviflora* and midstory trees are *Pandanus tectorius* and *Guamia mariannae*. Photograph by S.M. Boback.


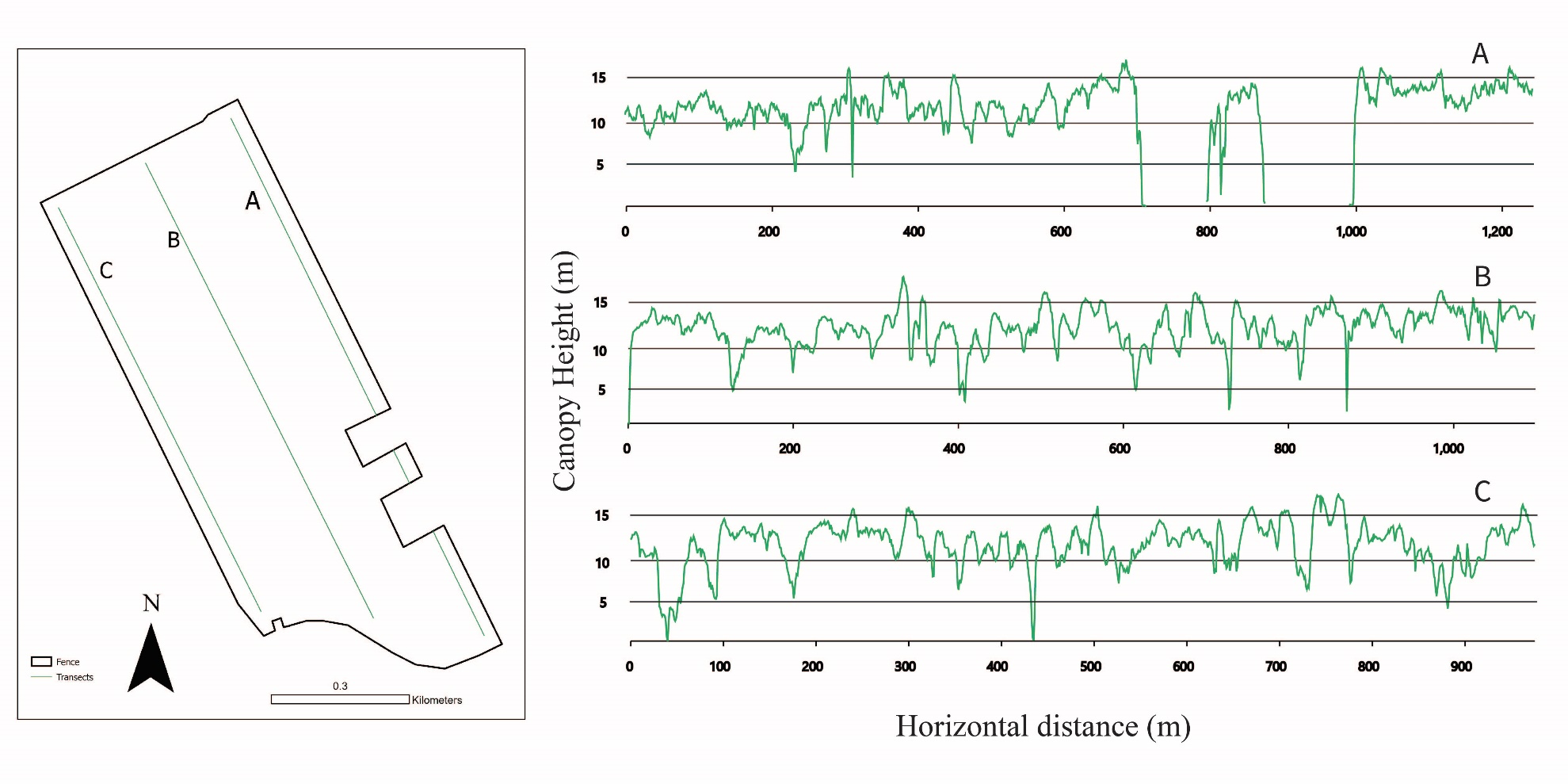


Figure S1


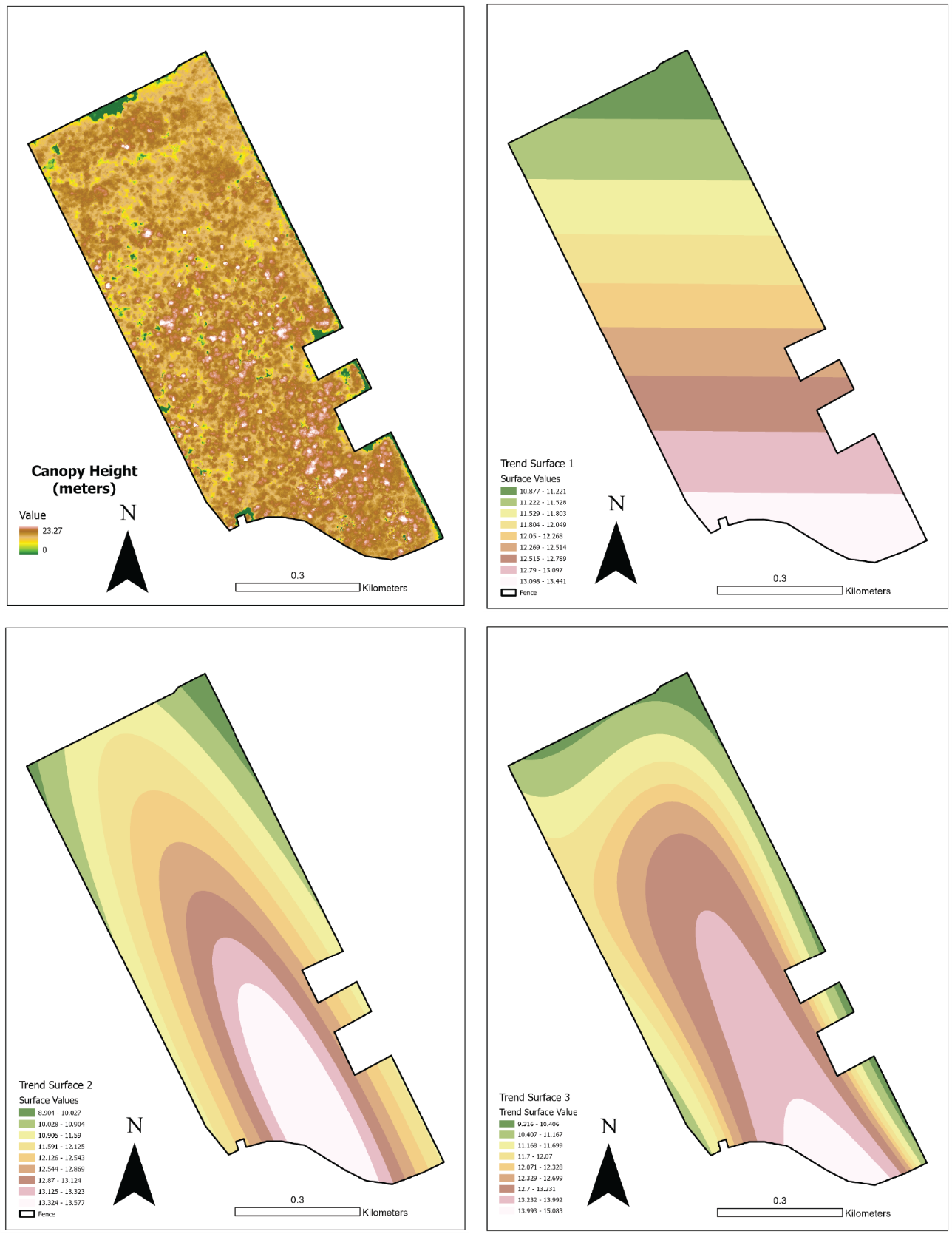


Figure S2


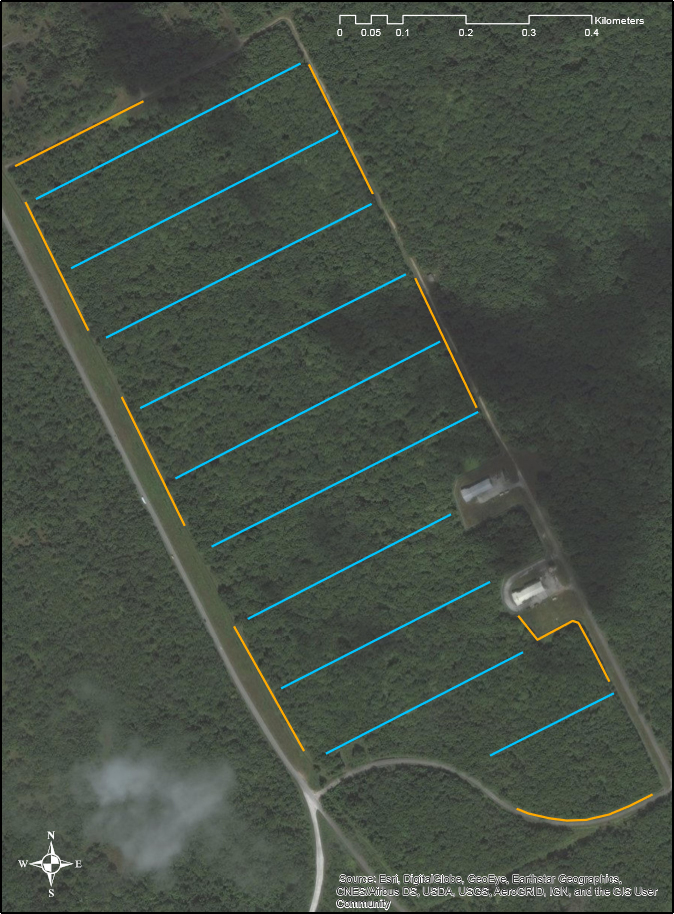


Figure S3


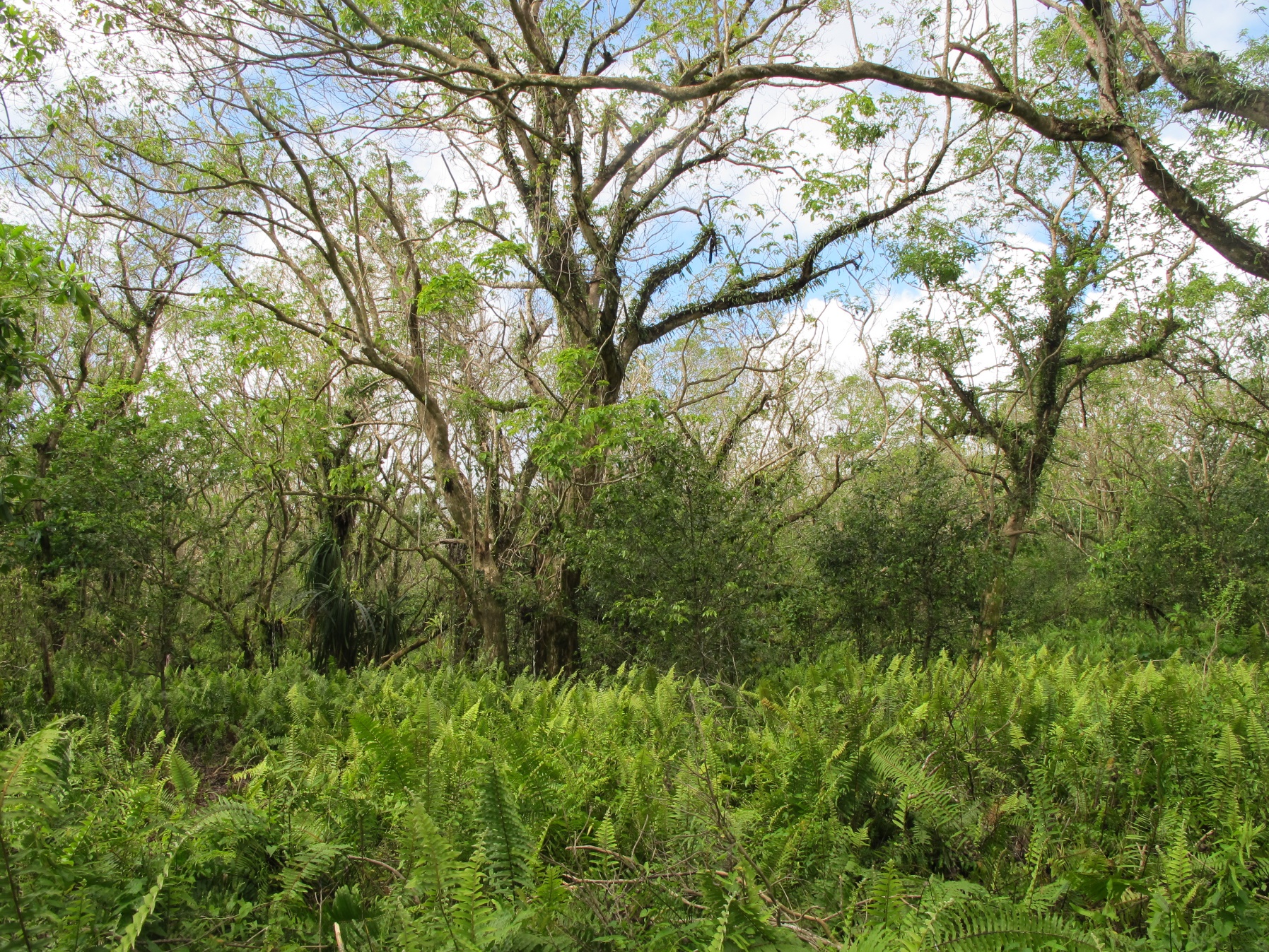


Figure S4

Table S1. Summary of movement parameters for 20 brown treesnakes (*Boiga irregularis*) radiotracked within the Habitat Management Unit (HMU) after snake suppression.

|  |  |  |  |  |  |  |  |  |  |  |  |  |  |  |  |
| --- | --- | --- | --- | --- | --- | --- | --- | --- | --- | --- | --- | --- | --- | --- | --- |
| **ID** | **Origin** | **Sex** | **SVL (mm)** | **Mass (g)** | **#Loc*** | **Days^1^** | **Dist^2^** | **Avg Dist^3^** | **Max Dist^4^** | **Max Disp^5^** | **Prop Vis^6^** | **Avg Ht^7^** | **MCP^8^** | **2D KUD^9^** | **3D KUD^10^** |
| 1 | Resident | F | 1150 | 210 | 56 | 121 | 3594 | 29.7 | 273.9 | 347.8 | 0.3 | 1.2 | 6.67 | 7.31 | 8.13 |
| 2 | Resident | F | 1161 | 195 | 54 | 121 | 3134 | 25.9 | 399.0 | 503.9 | 0.5 | 3.6 | 13.43 | 6.84 | 19.28 |
| 3 | Resident | F | 1172 | 261 | 57 | 122 | 1977 | 16.2 | 133.8 | 117.5 | 0.3 | 0.2 | 0.83 | 0.78 | 0.55 |
| 4 | Resident | F | 1225 | 424 | 61 | 142 | 2479 | 17.5 | 389.7 | 509.3 | 0.3 | 2.8 | 5.86 | 2.94 | 11.52 |
| 9 | Resident | F | 1045 | 179 | 51 | 114 | 2585 | 22.7 | 246.6 | 136.9 | 0.5 | 2.2 | 1.36 | 1.10 | 1.45 |
| 12 | Resident^†^ | F | 957 | 78 | 39 | 88 | 494 | 5.6 | 79.3 | 68.3 | 0.3 | 3.0 | 0.24 | 0.21 | 0.35 |
| 5 | Resident | M | 1604 | 755 | 56 | 123 | 6738 | 54.8 | 463.5 | 754.6 | 0.3 | 3.3 | 19.73 | 16.39 | 27.89 |
| 6 | Resident | M | 1214 | 271 | 51 | 114 | 2184 | 19.2 | 163.1 | 201.4 | 0.5 | 3.4 | 1.42 | 1.14 | 1.65 |
| 7 | Resident^R^ | M | 1820 | 1122 | 42 | 89 | 4369 | 49.1 | 452.4 | 469.6 | 0.5 | 1.5 | 13.39 | 9.72 | 18.53 |
| 8 | Resident | M | 923 | 103 | 50 | 114 | 1283 | 11.3 | 198.9 | 162.6 | 0.6 | 3.4 | 1.04 | 0.52 | 1.23 |
| 10 | Resident^R^ | M | 1114 | 182 | 30 | 52 | 1654 | 31.8 | 130.7 | 248.0 | 0.6 | 2.8 | 1.99 | 1.69 | 1.95 |
| 11 | Resident | M | 909 | 82 | 50 | 113 | 1321 | 11.7 | 102.4 | 130.3 | 0.5 | 2.6 | 0.66 | 0.44 | 0.92 |
| 22 | Resident | M | 1370 | 410 | 41 | 96 | 3937 | 41.0 | 233.5 | 181.0 | 0.5 | 2.8 | 4.77 | 4.88 | 8.06 |
| 14 | Translocated | F | 1023 | 163 | 56 | 122 | 2560 | 21.0 | 378.8 | 378.8 | 0.4 | 2.4 | 7.73 | 4.91 | 11.74 |
| 17 | Translocated^†^ | F | 986 | 124 | 50 | 114 | 3115 | 27.3 | 236.1 | 236.1 | 0.6 | 1.1 | 3.80 | 3.36 | 5.34 |
| 19 | Translocated | F | 954 | 125 | 50 | 115 | 2271 | 19.8 | 257.6 | 395.3 | 0.5 | 3.7 | 8.19 | 4.20 | 15.63 |
| 20 | Translocated | F | 1014 | 135 | 50 | 113 | 2368 | 21.0 | 419.6 | 419.6 | 0.5 | 1.8 | 11.92 | 2.88 | 8.62 |
| 21 | Translocated | F | 1016 | 125 | 49 | 114 | 1869 | 16.4 | 134.3 | 255.5 | 0.6 | 3.9 | 3.38 | 1.45 | 3.15 |
| 13 | Translocated^†^ | M | 1018 | 131 | 25 | 44 | 615 | 14.0 | 115.3 | 115.3 | 0.4 | 2.0 | 1.26 | 1.09 | 1.80 |
| 18 | Translocated | M | 982 | 122 | 50 | 113 | 1840 | 16.3 | 178.9 | 182.7 | 0.5 | 3.8 | 1.76 | 1.05 | 3.06 |
|  |  |  |  |  |  |  |  |  |  |  |  |  |  |  |  |
| *Total number of locations | | | |  |  |  |  |  |  |  |  |  |  |  |  |
| ^1^Total number of days tracked | | | |  |  |  |  |  |  |  |  |  |  |  |  |
| ^2^Total distanced moved in meters | | | |  |  |  |  |  |  |  |  |  |  |  |  |
| ^3^Average straight line distanced moved per day in meters | | | | | |  |  |  |  |  |  |  |  |  |  |
| ^4^Maximum distanced moved per day in meters | | | | | |  |  |  |  |  |  |  |  |  |  |
| ^5^Maximum dispersal distance from release site in meters | | | | | | |  |  |  |  |  |  |  |  |  |
| ^6^Proportion of time snake was visible to telemetry teams | | | | | | | |  |  |  |  |  |  |  |  |
| ^7^Average perch height in meters | | | | |  |  |  |  |  |  |  |  |  |  |  |
| ^8^Activity area as measured by Minimum convex polygon method in hectares | | | | | | | | | |  |  |  |  |  |  |
| ^9^2D Activity area as measured by 2D Kernel Utilization distribution method in hectares | | | | | | | | | | |  |  |  |  |  |
| ^10^3D activity volume as measured by 3D Kernel Utilization distribution method in cubic hectares | | | | | | | | | | | |  |  |  |  |
| ^†^Animal died prior to termination of study | | | | | |  |  |  |  |  |  |  |  |  |  |
| ^R^Transmitter failed before termination of study | | | | | |  |  |  |  |  |  |  |  |  |  |

Table S2 Movement patterns for 9 transmittered brown treesnakes (*Boiga irregularis*) within the Habitat Management Unit (HMU) immediately prior to, and after observing evidence of prey consumption (presence of a noticeable prey bulge). Apart from the observation on 7/9/2015 when a BTS was observed swallowing a shrew, all estimates in this table are based upon the first date where evidence of prey consumption was observed and not the precise day of ingestion.

|  |  |  |  |  |  |  |  |  |
| --- | --- | --- | --- | --- | --- | --- | --- | --- |
|  |  |  |  | **DISTANCE MOVED (M)** | | | | |
| **Snake #** | **Sex** | **Date of observation** | **Evidence of prey consumption** | **Day prior** | **Day of observation** | **Day after** | **Two days after** | **Avg daily move** |
| 1 | F | 7/7/2015 | prey bulge | 79.6 | 96.8 | 18.7 | 24.3 | 29.7 |
| 2 | F | 6/16/2015 | prey bulge | 24.4 | 74.6 | 4.5 | 66.7 | 25.9 |
| 2 | F | 7/29/2015 | prey bulge | 22.9 | 26.3 | 22.0 | N/A | 25.9 |
| 3 | F | 6/23/2015 | prey bulge | 52.0 | 58.0 | 68.1 | 133.8 | 16.2 |
| 8 | M | 7/14/2015 | prey bulge | 47.8 | 3.3 | 7.3 | 4.9 | 11.3 |
| 9 | F | 7/28/2015 | prey bulge | 40.1 | 81.1 | 15.5 | 2.2 | 22.7 |
| 10 | M | 7/13/2015 | prey bulge | N/A | 17.5 | 45.5 | 115.0 | 31.8 |
| 13 | M | 6/23/2015 | prey bulge | 19.2 | 18.4 | 8.8 | 3.9 | 14 |
| 13 | M | 7/9/2015 | consuming shrew | 16.8 | 10.0 | N/A | N/A | 14 |
| 18 | M | 6/16/2015 | prey bulge | 126.9 | 14.1 | 13.6 | 7.9 | 16.3 |
| 21 | F | 6/17/2015 | prey bulge | 82.9 | 4.9 | 5.5 | N/A | 16.4 |
| 21 | F | 6/25/2015 | prey bulge | 15.3 | 3.5 | N/A | N/A | 16.4 |
| 21 | F | 7/1/2015 | prey bulge | N/A | 36.0 | 3.9 | N/A | 16.4 |
