# Supplementary figures and images for: Invasive brown treesnakes (Boiga irregularis) move short distances and have small activity areas in a high prey environment
Source: Sci Rep. 2022 Jul 26;12:12705. doi: 10.1038/s41598-022-16660-y (PMC9325984; doi:10.1038/s41598-022-16660-y)

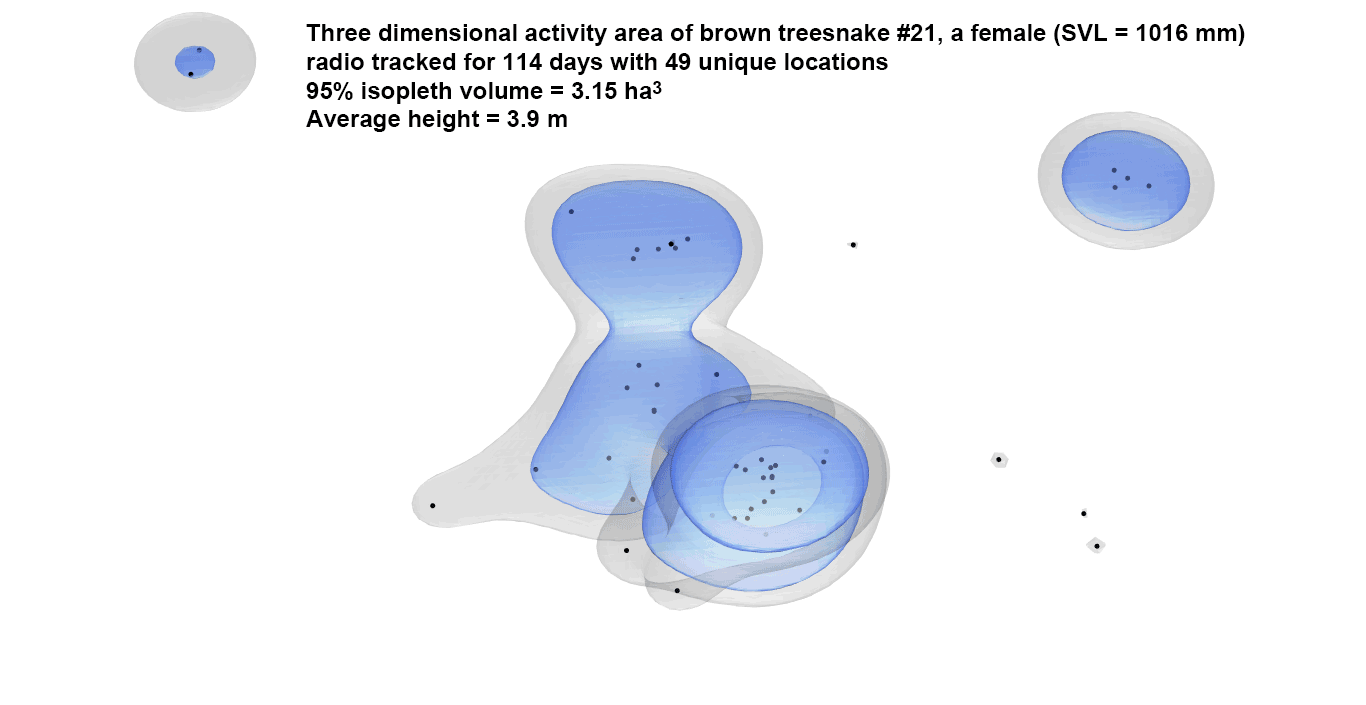

Supplement: Supplementary file 2 — Supplementary Video S1. [file 41598_2022_16660_MOESM2_ESM.gif]

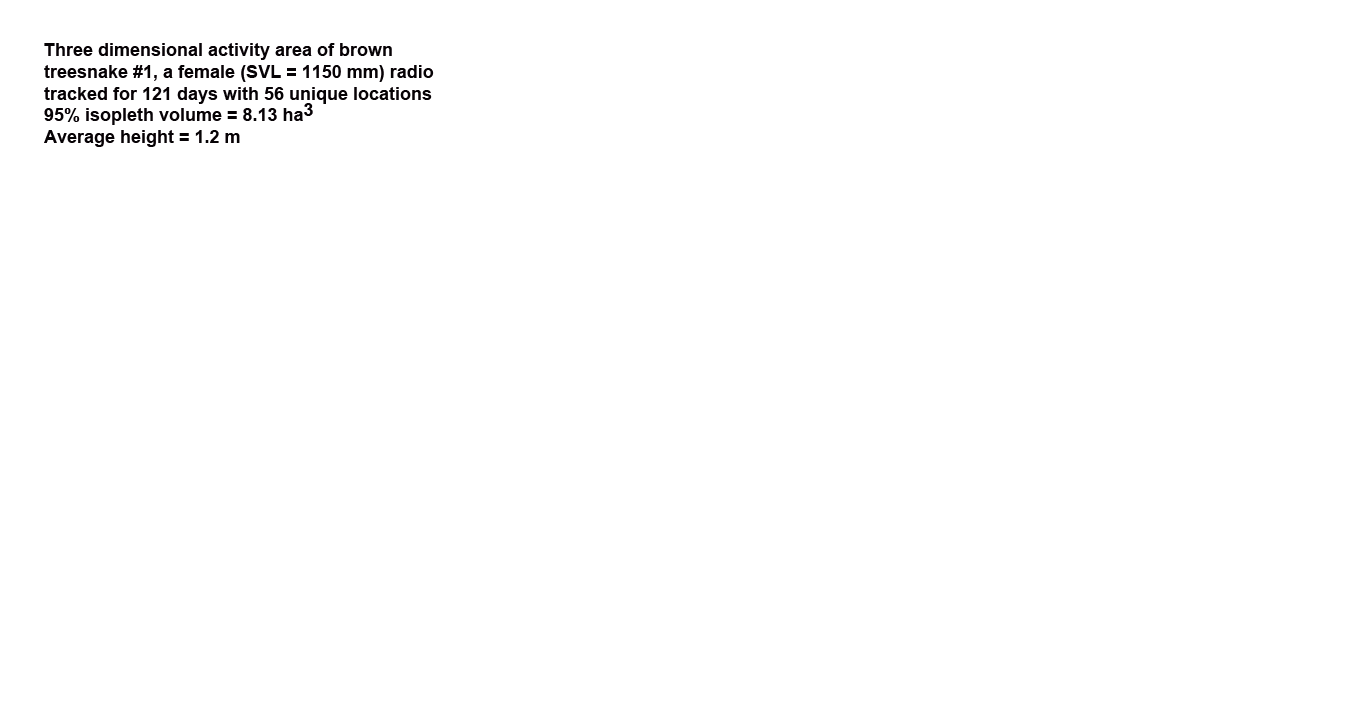

Supplement: Supplementary file 3 — Supplementary Video S2. [file 41598_2022_16660_MOESM3_ESM.gif]
